# Exploring Hydrochars from Lignocellulosic Wastes as Secondary Carbon Fuels for Sustainable Steel Production

**DOI:** 10.3390/ma16196563

**Published:** 2023-10-05

**Authors:** Álvaro Amado-Fierro, Teresa A. Centeno, María A. Diez

**Affiliations:** Institute of Science and Technology of Carbon (INCAR), CSIC, Francisco Pintado Fe 26, 33011 Oviedo, Spain; alvaro.af@incar.csic.es (Á.A.-F.);

**Keywords:** coal injection, biomass, biowaste, charcoal, hydrochar, PCI blends, pyrolysis, combustion, gasification, blast furnace

## Abstract

This study investigates the suitability of different lignocellulosic sources, namely eucalyptus, apple bagasse, and out-of-use wood, for injection into blast furnaces (BFs). While wastes possess carbon potential, their high moisture renders them unsuitable for direct energy utilization. Additionally, the P and K impurities, particularly in apple bagasse, can pose operational and product quality challenges in BF. Thus, different thermochemical processes were performed to convert raw biomass into a more suitable carbon fuel. Low-temperature carbonization was selected for eucalyptus, yielding a biochar with properties closer to the low-rank coal. Hydrothermal carbonization was chosen for apple bagasse and out-of-use wood, resulting in hydrochars with enhanced fuel characteristics and fewer adverse inorganic species but still limiting the amount in binary PCI blends. Thermogravimetry evaluated the cause–effect relationships between coal and coal- and bio-based chars during co-pyrolysis, co-combustion and CO_2_-gasification. No synergistic effects for char formation were observed, while biochars benefited ignition and reactivity during combustion at the programmed temperature. From heat-flow data in combustion, the high calorific values of the chars were well predicted. The CO_2_-gasification profiles of in situ chars revealed that lignin-rich hydrochars exhibited higher reactivity and conversion than those with a higher carbohydrate content, making them more suitable for gasification applications.

## 1. Introduction

Coal is the primary carbon and energy source in iron and steel production, used in coking plants to produce coke for blast furnaces (BF) and in coke-based BFs using pulverized coal injection (PCI). This reliance on coal contributes significantly to the industry’s substantial CO_2_ emissions (2.6 Gt annually) and high energy consumption [1]. CO_2_ emissions from the BF–BOF route average 1.83 tons per ton of crude steel, mainly from iron ore reduction reactions [2]. With global steel demand projected to rise by one-third by 2050, the industry faces a pressing need to reduce its carbon footprint through innovative technologies [1,2,3,4,5,6,7,8,9,10,11,12,13,14]. In the near-term Sustainable Development Scenario, substituting coal with less carbon-intensive materials like thermally treated biomass, charcoal, plastics, and oils (with higher H/C atomic ratios) offers sustainable alternatives while maintaining competitiveness [1,2,3,4,5,6,7,8,9,10,11,12,13,14].

Pulverized coal injection (PCI) into BFs shows promise for introducing biomass and other renewable carbon materials as alternatives to mitigate fossil CO_2_ emissions. However, not all biomass types are suitable for direct injection, necessitating thermochemical conversion processes like carbonization, torrefaction, or hydrothermal carbonization to enhance physical and chemical properties. Upgraded woody biomass, such as charcoal, offers advantages over coal, including a lower ash and sulfur content [3,15,16,17,18]. Charcoal is already used in BFs commercially in Brazil [15,19]. Nonetheless, challenges remain in scaling up biomass use due to the lower mechanical strength of charcoal, biomass availability, and the need for further charcoal production improvements [1].

For a technical implementation of other types of biofuels in an environmentally and economically efficient manner, in-depth research is needed to assess the effective use of biowastes as valuable secondary fuels in a BF or other industrial applications. Raw biowaste is not directly usable for BF operations as woody biomass, and some pretreatment is also needed. For instance, in the Torero project developed by ArcelorMittal [10], torrefaction is the thermochemical process selected to convert waste biomass into a coal-like material, replacing coal for injection into the blast furnace. The source material is waste wood, typically from demolition sites, that is currently collected and incinerated [10]. Future projects will evaluate other waste carbons like agricultural and forestry residues and even waste plastics [10].

In the present study, hydrothermal carbonization (HTC) is selected for the pretreatment of wet biowastes because of their low cost and environmental concerns [20]. HTC is also a thermochemical process at low temperatures ranging from 180 to 250 °C, giving a more versatile and stable carbon-rich material (hydrochar) than conventional lignocellulosic biomass. Under these conditions, this technology allows for saving energy costs. Along with the solid product with a typical yield of 45–70% by weight of the initial wet biomass, an aqueous stream accounting for 5–25% is also generated, while the gases released are limited to 2–5% [21]. Depending on the severity of the treatment, hydrochars are like peat and lignite and, in more extreme cases, like sub-bituminous coal or hard lignite. 

Due to the high explosiveness, several studies have reported that hydrochar alone is unsuitable for direct injection in the blast furnace [20,21,22,23]. Combining hydrochar with charcoals or coals may decrease CO_2_ emissions and reduce coal consumption. So, it is recommended to mix the studied hydrochar from paper mill biological sludge with anthracite, not exceeding 30% hydrochar addition. This strategy reduced the ignition point of anthracite and improved its ignition and combustion performance [21]. It was also found that hydrochars from different biogenic sources, like maize and wheat straw, in co-combustion with anthracite, decrease the ignition and burn-out temperatures by increasing the hydrochar blending ratio [23]. However, the best addition ratio is as high as 60% due to a catalytic role during combustion. The combustibility improved due to the looser pore structure, higher alkali metal content in the ash, more active functional groups in the C-network and lower ordering degree, equivalent to low-rank coal [23].

Biowaste like lemon peel has also been investigated for hydrochar production [22]. At a high temperature of 1200 °C, only 54% of carbon remained in the solid product. Consequently, the amount of carbon available for high-temperature reactions, such as combustion, reducing gas generation, carburization, and slag foaming, is significantly reduced. The hydrochar resulted in a less-efficient material compared to charcoal and anthracite. In contrast, the advantages of hydrochar were its lower ash and S concentrations and renewability.

The extent to which hydrochar can be blended with injection coals/charcoals and the effect of source material or treatment severity on blast furnace operation is still unclear. In the present work, a systematic approach was carried out to evaluate the potential of various hydrochars obtained from biowastes like out-of-use wood and apple bagasse by HTC at 180 °C and 230 °C for 2 hours. The study described preliminary results comparing the chemical characteristics and thermal behavior of hydrochars from lignocellulosic wastes and chars from eucalyptus and coal for blending with PCI coal under different atmospheres, inert and reactive, for the effective screening of potential renewable carbon fuels as secondary components in blends. Pyrolysis, combustion and CO_2_-gasification of the single fuels (coal and biomass) and blends with low-volatile coal were performed using thermogravimetric analysis to gain information on (1) the organic composition and the transformations experienced during the HTC process and in situ char formation; (2) the characteristic parameters of single components and blends as fuel during combustion; (3) the CO_2_-gasification of the unburnt char; and (4) how the different carbon sources interact in blends during the thermochemical conversion processes. The methodology combines the thermal behavior in the three environments that could make an early classification of new lignocellulosic materials as heat sources and carbon reductants according to their similarity to conventional fuels (coal, PCI blends, and coke).

## 2. Materials and Methods

### 2.1. Coal and Raw Biomass

Low-volatile bituminous coal (LVC) with a volatile matter of 18.4 wt% db was used as the base coal for blending with two groups of coal- and biomass-based materials. The first group includes one high-volatile coal (HVC), *Eucalyptus globulus* (EU), and their corresponding chars (CHV and CEU) obtained at 450 °C. The coal HVC and its char (CHV) were used as reference secondary fuels. *Eucalyptus* was selected for its rapid growth, wide adaptability to different soil qualities and climates, and widespread use of its charcoal in mini-BF [15]. This group was selected to compare the chemical characteristics and thermal behavior with a second group that contains two lignocellulosic wastes, apple bagasse (AB) and out-of-use woods (OW), and their laboratory-prepared hydrochars. AB is a waste from apples hydraulically pressed for manufacturing natural cider by a local cellar (Llagar Fanjul, Siero, Asturias-Spain). It is primarily composed of the skin, seeds and pulp of apples and is usually intended for animal feed, composting, incineration and landfilling. The other waste used, OW, was provided by the Company for Waste Treatment of the Company for Solid Waste Management in Asturias (COGERSA, Asturias, Spain). The OW waste that ends up in landfills comes from furniture and building demolition; substances such as paints and oils are expected to be found as minor foreign components. The biomass wastes were subjected to natural drying by forced air circulation at 105 °C for 48 h to reach a residual moisture content of less than 5 % by weight for subsequent analysis.

One metallurgical coke with a moderate CO_2_ reactivity index (CRI) of 25.4%, high post-reaction mechanical strength (CSR) of 64.6%, and a calorific value of 29 MJ/kg was included as reference carbon material for the blast furnace.

### 2.2. Low-Temperature Pyrolysis

Pyrolysis experiments with HVC and EU (5 g with a particle size <1 mm) were performed in a horizontal electrically heated oven at 5 °C/min to a final temperature of 450 °C, with a soaking time of 15 min under an atmosphere of evolving gases. The condensable products (water, light oil and heavy oil or tar) obtained during the carbonization experiments were collected employing a NaCl-ice-cooled trap (oil/tar yield: 43.4%). The non-condensable gas yield was estimated by difference (gas yield: 18.7%). The biochar yield was 37.9%. The mass balance was calculated on a dry basis relative to the starting material.

### 2.3. Hydrothermal Carbonization (HTC)

Two target temperatures were selected to produce the hydrochars, 180 and 230 °C, maintaining a soaking time of 2 h. The water/dry biomass ratio was 4:1, considering the water already existing in the solid wastes. HTC experiments were carried out in a 3 L stainless steel autoclave equipped with a heating mantle on the outside and a refrigeration circuit capable of cooling down the sample if needed. The sample temperature and the pressure inside the reactor (1.2–2.9 MPa) were measured. After each carbonization, the hydrochar and the liquid phase were separated through filtration. Then, hydrochar was rinsed with 1500 mL of water to remove the remains of the liquid phase. Hydrochar was finally dried in an oven at 105 °C for at least 24 h, weighed to determine the yield and crushed for subsequent analyses. The identification code of the resulting hydrochars consisted of 4 alphanumeric characters, indicating the raw biowaste (AB, OW) followed by two numbers identifying the HTC temperature (1 and 2 for 180 and 230 °C, respectively) and the second digit (2) indicated the soaking time. 

### 2.4. Characterization of Raw and Thermal-Treated Coal and Biomass

Proximate analysis was performed to classify the fuels by their thermochemical compositions (moisture, volatile matter, and ash contents). After drying and devolatilization, the ash-free carbon-rich solid (fixed carbon) was calculated by subtracting the percentages of the moisture, ash and volatile matter from 100%. 

The elemental analysis was carried out using a LECO CHN-2000 for C, H and N (ISO 29541 [24]), and a LECO S-144 DR (LECO Corporation, St. Joseph, MO, USA) was employed for the total sulfur content (ISO 19579 [25]). The oxygen content was calculated from the mass balance on a dry basis.

The higher heating value (HHV) was evaluated using an IKA C4000 adiabatic bomb calorimeter (IKA Analysentechnik, Heitersheim, Germany), and the lower heating value (LHV) of all the samples was calculated according to the ISO 1928:2009 [26] standard procedure. The HHV was also calculated using the mathematical model developed by Channiwala and Parikh [27] and based on elemental analysis of the fuels. The experimental HHV values agree with the calculated values; the deviation being less than 3%.

The composition of ashes obtained at 815 °C by air oxidation was determined using X-ray fluorescence (XRF) using a Siemens-Bruker SRS3000 spectrometer (Siemens SRS 3000, Siemens, Munich, Germany, now: Bruker AXS GmbH, Karlsruhe, Germany), according to the ASTM D4326-04 [28] standard procedure. The major elements analyzed were used to define the basicity or alkali index (BI), based on the ratio between the basic and acid components in the ashes (A) and can be expressed as

(1) $$\mathrm{BI}=A\;\frac{\left({\mathrm{Na}}_{2}O+{\;K}_{2}O+\mathrm{CaO}+\mathrm{MgO}+{\mathrm{Fe}}_{2}O_{3}\right)}{\left({\mathrm{SiO}}_{2}+{\;\mathrm{Al}}_{2}O_{3\;}+{\;\mathrm{Ti}}_{2}O\right)}$$

### 2.5. Thermogravimetric Analysis (TG/DTG)

The single fuels and binary blends were subjected to thermogravimetric analysis under inert (N_2_), oxidizing (air), and reactive (CO_2_) atmospheres. 

Pyrolysis was performed dynamically from 30 °C to 800 °C at a 20 °C/min heating rate, with a soaking time of 10 min and a nitrogen flow rate (inert and sweep gas) of 75 ml/min. 

Combustion was carried out under the same heating conditions in a 75 ml/min air flow, while CO_2_-gasification tests consisted of a first pyrolysis step in dynamic mode from 30 °C up to 800 °C at 20 °C/min under an N_2_ atmosphere to produce the char and a CO_2_-gasification of the char from 750 °C to 1000 °C, followed by an isothermal step at 1000 °C for 2 h.

Representative samples of about 10 mg with a particle size lower than 212 μm (the same particle size used in conventional fuel analysis) were evenly distributed in an open platinum pan. All TG experiments were conducted in a Mettler Toledo TGA/DSC1 Star thermoanalyzer (TA Instruments, Greifensee, Switzerland). Weight loss and heat flow (in milliwatts) were recorded simultaneously as a function of time and temperature, and the first derivative of the weight loss over time (DTG) was calculated. 

## 3. Results and Discussion

### 3.1. Characteristics of Raw Materials and Thermochemical Conversion Products

A comparison of the main characteristics of the raw materials and the coal- and bio-based chars is shown in Table 1. The two coals selected, LVC and HVC, had different ranks, with volatile matter content of 18.4 and 37.8 wt% db, respectively, and sulfur and ash contents below the preferred limits for injection in BFs [13]. Both coals are often blended to optimize their composition and thermal behavior, reduce raw material costs, and dilute unfavorable characteristics of the components. The selected lignocellulosic materials were characterized by a high volatile matter (79.0–83.7 wt% db) and low ashes (0.16–2.30 wt% db), resulting in low fixed carbon (16.1–18.7 wt% db) compared to the two coals (56.5 and 70.9 wt% db for HVC and LVC coals, respectively). 

Biomass, a composite material made up of three structural oxygen-rich biopolymers (hemicelluloses, cellulose and lignin), had a high oxygen content ranging between 42 and 46 wt% db and a low C content (about 46 wt% db), leading to much lower HHV and LHV than those of the low-rank coal HVC. One peculiarity of the wastes, the apple bagasse (AB) and out-of-use wood (OW), was the excessively high-water content, particularly for AB waste, rendering these raw wastes inadequate for energy. 

The ash composition also contributes negatively to energetic applications. In particular, AB has unacceptable amounts of phosphorus and potassium in the ashes (Table 2). For BF, such ash-forming elements, phosphorus and total alkalis (Na + K) in coal and coke must be kept as low as possible because phosphorus entering the BF ends up in the hot metal, affecting its quality. Potassium causes BF operating problems; it recirculates around the furnace before leaving either in the top gas or gradually builds up in the lower BF regions and, finally, in the slag. When deposited on the walls, it significantly impacts the lining life and furnace stability, and it also chemically attacks the carbon structure, accelerates carbon gasification and weakens the coke. The significant sources of P and alkaline elements (Na and K) in BFs come from the ashes of coal, coke, and the gangue materials in the ore, making it impossible to eliminate and limiting the quantity in the inputs is the only way to control the adverse effects [12]. Although the optimum values for injectants strongly depend on the characteristics of BF and operational conditions, some indicative PCI coal specifications from different plants may have a desirable P amount, ranging from 0.01 to 0.06 wt% maximum, and alkali oxides (Na_2_O plus K_2_O) below 0.35 wt% [12,13], and similar levels were fixed for metallurgical coke [29,30,31,32].

Regarding the ash composition, other highlights can be drawn. All biomass ashes contained significant amounts of CaO, ranging between 9 wt% and 25 wt%, which were relatively higher than the quantities in coal ashes. Basic-Ca species can improve the BF reaction efficiency by reducing the fusion temperature and viscosity and acting as an effective catalyst in the gasification reaction of unburnt char. Additionally, eucalyptus ashes possess a predominance of iron that can significantly promote gasification reactivity.

In summary, from the chemical composition and thermal energy capacity—in terms of the P and K, moisture, volatile matter, more oxygen than C and H, and low calorific value—it is evident that the non-C species in the raw wood and biomass wastes are extremely high. Therefore, the fixed carbon and calorific value are too low to be used as fuel, although there may be other reasons for their utilization, mainly when waste materials are recovered in compliance with local environmental standards. In this context, pretreatments are required to produce a fuel with better specifications and higher energy densities closer to the PCI coals. Among the different processes for the optimal thermochemical conversion of raw biomass into an energetically valorized product, the chemical composition largely determines the process selection, with water content and ash composition influencing the choice of conversion pathway. Thus, slow carbonization at a low temperature was the selected process for eucalyptus wood. It yields around 38 wt% db of biochar of the type of charcoal (CEU), increasing the C content and HHV to levels closer to PCI coals and the char produced from the high-volatile coal (CHV) under the same conditions. As regards the raw lignocellulosic wastes, AB and OW, and due to the high-water levels, hydrothermal carbonization (HTC) was selected since such a process may significantly improve the characteristics of the organic and inorganic fractions of the low-grade biomass while avoiding the need for a drying pretreatment [33].

Furthermore, HTC may provide an attractive approach to expanding non-fossil carbon resources for ironmaking if such wastes could be valorized to added-value products that meet fuel selection criteria. Applying the HTC process at two different temperatures, 180 and 230 °C, with 2 h soaking time, the water content of the hydrochars can be reduced by drying up to desirable levels (Table 1), enhancing the handling and storage stability and producing a variation of the composition towards more thermally stable chemical constituents. The yield of solid hydrochars was 73 and 83 wt% on a dry basis at 180 °C, and 62 and 61 wt% at 230 °C for AB and OW, respectively.

Devolatilization of the precursors occurs, increasing carbon, substantially decreasing oxygen and giving a more carbon-enriched structure. Accordingly, the HHV increases to values between 20.8 and 28.9 MJ/kg, which fits into the coalification region of lignite and high-volatile bituminous coals [34]. 

**Figure 1:**
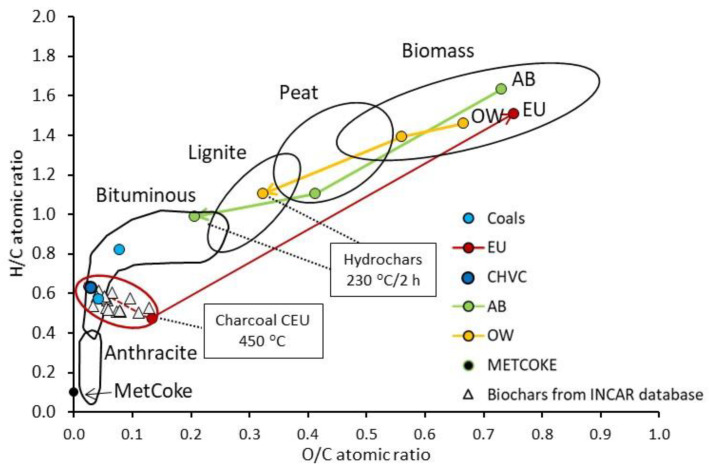
Classification of raw materials and coal- and bio-based chars in the van Krevelen diagram.

The degree of the compositional changes is linked to the temperature applied and the precursor treated. Devolatilization is higher for apple hydrochars than for out-of-use wood hydrochars, implying more remarkable structural and compositional modifications under the same conditions, probably due to the water acting as solvent and catalyst [35] and to a higher quantity of thermally unstable constituents at low temperature contained in the precursor. All these changes are better visualized by plotting the O/C and H/C atomic ratios in the van Krevelen diagram (Figure 1). As evidenced by elemental composition, apple hydrochars have higher development than out-of-use woods, reaching a bituminous coal-like state at the harshest conditions, while the HTC applied to OW gives products with still high oxygen content.

Based on these results, the out-of-use wood cell wall seems more resistant to the subcritical-water action and may need harsher conditions for a more remarkable evolution of the cell wall network. Nevertheless, compared to eucalyptus charcoal produced at relatively low-temperature and short residence time, the two hydrochars are still in the earlier region of coalification degree. Figure 1 also shows an upward trend with decreasing O/C-increasing H/C from eucalyptus biochar towards the char from high-volatile coal (CHV) and the coalification region of LVC coal. 

Moreover, there are more benefits to the HTC valorization of the wastes; not only is the organic fraction of the biomass feedstocks modified by the process but several differences in the ash composition of the solid products can also be observed. The two most abundant elements in raw apple bagasse are K and P, which account for nearly 65% of ashes, whereas in out-of-use-woods are Si and Ca (Table 2).

**Figure 2:**
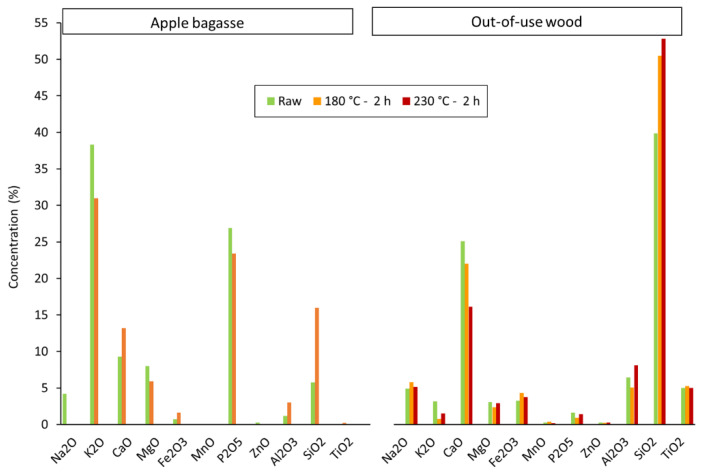
Ash elemental composition of apple bagasse and out-of-use woods and their respective hydrochars by XRF.

HTC treatment of AB at low severity partially eliminates the harmful K and P elements (Figure 2). Additionally, Na is practically removed as a significant element in the ashes (4.2% in AB vs. 0.01% in AB12), contributing to decreased alkali oxides. On the contrary, there is an increase in the presence of Si and Ca. Consequently, some inorganic species are generally solubilized by HTC, reducing the total ashes to less than 1 wt% compared to the parent material (Table 1).

In the case of out-of-use woods, Si levels are further increased at the harshest conditions, whereas Ca levels are reduced due to the partial solubilization of some Ca-based mineral species during HTC (Figure 2). Foreign constituents, such as titanium from paint chemicals in the original OW sample, are maintained at the same level (5%).

Insights into the thermal behavior and how the different fuels interact with coal under different atmospheres (inert and reactive), pyrolysis, combustion and CO_2_-gasification of coals, raw biomass, and chars as single fuels are provided by thermogravimetric analysis. To overcome the inherent limitations of using biomass compared to coal, a series of binary blends with LVC as the base PCI coal and the different chars were prepared. The blend containing 20 wt% of the high-volatile coal (HVC) is used as an injection reference blend. Based on the ash chemistry of AB12 hydrochar, the replacement percentage ratio in the PCI blends was calculated as 10 and 20 wt% to achieve the BF input requirements (P, 0.04 wt% and alkali oxides 0.27 wt%). The limitation of high P and alkalinity in hydrochar ash has been reported by other authors, exploring the use of hydrochars from different feedstocks, e.g., lemon peels treated under a pressure of 2 MPa and an HTC temperature of 210 °C, for blending with anthracite in the steel production by different routes [18].

### 3.2. Pyrolytic Behavior Using Thermogravimetry

**Figure 3:**
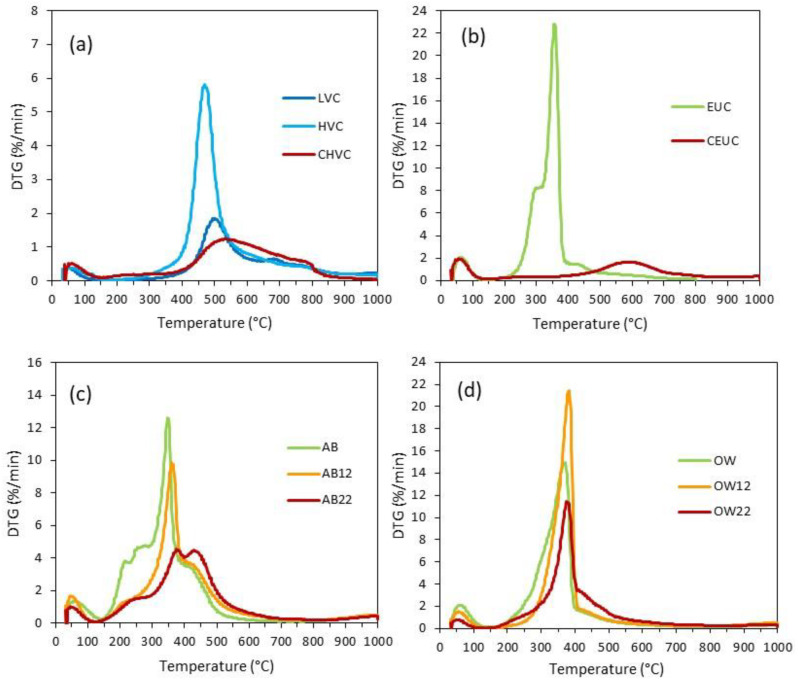
Pyrolysis DTG curves of the raw materials and respective chars of coals (**a**), eucalyptus (**b**), apple bagasse (**c**) and out-of-use woods (**d**).

Using thermogravimetry under pyrolysis conditions, the distribution of volatiles evolved during char formation as a function of temperature can be quantitatively evaluated. Figure 3 shows the DTG profiles of the coals, raw biomasses, and the resultant coal- and bio-based chars. Additionally, Table S1 summarizes some relevant parameters derived from the thermogravimetric analysis. The evolution of volatiles of the two coals corresponds to their rank. The HVC coal evolves the volatiles at a greater rate, DTG_max_ of 5.81 %/min, and a lower temperature (470 °C vs. 494 °C), starting around 325 °C, ending nearly to 990 °C and being the rate more gradual from 600 °C. Maximum weight loss occurs between 400 and 600 °C, and the quantity of volatile matter evolved is about 25.3 wt% in this temperature interval. 

Regarding the char yield linked to the coal rank, a lower yield is obtained for HVC (63.7 vs. 80.6 wt%). The resultant CHV char at a laboratory scale exhibits a progressively less intense devolatilization in a broad temperature interval from 400 to 800 °C due to cracking and polymerization reactions of the C-network, as it is also seen for eucalyptus charcoal, with T_max_ occurring at around 530 °C, and a char yield increases up to 79 wt% at the highest temperature.

Raw eucalyptus exhibits a typical DTG pattern of hardwoods where the cellulose coexists with hemicelluloses and lignin in complex physical and chemical associations within the cell wall [36,37]. In biomass wood resources, cellulose is the most abundant structural biopolymer (39–54 wt%), with hemicelluloses (20–30 wt%) surrounding the cellulose fibers and as a link between cellulose and lignin. The latter is a cross-linked phenolic polymer, which accounts for 19 to 26 wt%. Generally, hemicelluloses have a much lower molecular weight than cellulose; some are branched and soluble in alkali, easily hydrolyzed by acids, and less thermally stable [38]. Non-structural organic compounds of different chemical classes (terpenes, proteins, tannins, fats and waxes) in various amounts (4–10 wt%) are also in wood. They are soluble in organic solvents or water (extractives) and are generally less thermally stable than structural biopolymers.

Concerning the thermal decomposition of such woody components, after water evaporation, structural transformation starts and ends at about 200 and 800 °C, respectively, and reaches a maximum devolatilization at around 355 °C. The peak at DTG_max_ is attributed to cellulose degradation, which reaches its maximum decomposition rate after the degradation of extractives and hemicelluloses, the breakdown of β-O-4 lignin bonds (more thermally unstable in less mature lignin), and hemicellulose/lignin connections (peak fronting at 300 °C) [37]. Depending on the lignin maturity and thermal stability, lignin slowly breaks down over a wide temperature range, 200–800 °C, and tails off above 430 °C in the DTG profile (flat tailing peak). Compared to the precursor, CEU char exhibits a drastic change in DTG, and it undergoes cracking and polymerization reactions beyond 400 °C up to nearly 800 °C, producing a low and wide volatile evolution that moves towards much higher temperatures (T_max_ at 580 °C and DTG_max_ of 1.98 %/min). The partial overlapping of the volatile matter emission from the chars of eucalyptus and HVC, in the 400–800 °C range, is consistent with the elemental and proximate composition similarity (Table 1). Considering that the HVC coal is used for PCI blending, the eucalyptus biochar seems to have great potential to substitute HVC coal. 

Attending to apple bagasse and out-of-use wood, the pyrolysis of the starting wastes also follows the typical DTG pattern of lignocellulosic biomass [36,37]. Without considering the peak associated with water, hydrochars present significant changes at 150–300 °C, more pronounced at the more severe HTC conditions: reduction of extractives, hemicelluloses due to an acid-hydrolysis in the reaction medium to oligomers and monomers, and the less mature lignin that is easier to degrade. Highly condensed lignin that is more easily converted into char remains. All the composition modifications are associated with diminishing the volatiles evolved below 400 °C, and the increase detected at a much higher temperature (Table S1). 

In the case of out-of-use woods (Figure 3), the DTG profile is similar to that of eucalyptus wood, although differences in the compositional distribution of structural and non-structural components bring some differences. The presence of extractives seems less pronounced, and the hemicelluloses are hardly detected (peak fronting at around 300 °C). For this waste, a mild HTC treatment appears to increase the DTG_max_ when extractives and hemicelluloses are partially removed, and cellulose decomposition occurs at a higher temperature with an increase in DTG_max_ (21.43 vs. 14.92 %/min). On the contrary, a more severe HTC treatment also causes a relative decrease in cellulose degradation, an increase in the lignin feature and an enhancement of hydrochar yield up to 35.6 wt%. The HTC process could be a promising recycling alternative to conventional waste disposal, contributing to new, high-value products tailored for a given application.

For AB, a different low-temperature pyrolysis pattern can be distinguished, which is associated with the compositional distribution of structural and non-structural components of this fruit processing waste. A primary peak at around 350 °C, attributed to cellulose decomposition, exhibits a maximum DTG of 12.63 %/min, with two partially overlapped shoulders with maximum decomposition rate at 212 and 280 °C associated with a mass loss of around 25 wt% and a last event after cellulose peak, which agrees with a high content of more mature lignin. AB is a pectin-rich lignocellulosic biomass containing around 20 wt% of the hetero-polysaccharides class with a core of α-1,4-D-galacturonic acid and several neutral sugars such as arabinose, galactose, among others [39]. The lowest temperature shoulder at 212 °C is evident in this waste and is associated with the cleavage of the weakest C–O bonds of the acid and ester side groups in pectins [40]. A second shoulder follows it between 250 and 300 °C, linked to hemicellulose degradation. Organic extractives also contribute to the volatile emission of up to 300 °C. The amount of extractives in this type of fruit-waste is also high, about 24 wt%; cellulose only accounts for nearly 18 wt%, and hemicellulose and lignin join the different layers of cell walls are around 10 and 25 wt%, respectively [39].

One consequence of HTC treatment in AB is the delay of the T_max_ attributed to cellulose and the decrease in the volatilization rate. Thus, as HTC conditions become more extreme, cellulose seems to be considerably affected and more modified in the hydrochar from apple bagasse at 230 °C (AB22). The reduction in conversion rate manifests the enhanced thermal stability of hydrochars over raw biomass, which varies considerably depending on the pyrolysis temperature (a decrease in volatiles evolved at 200–300 and 300–400 °C, with an increase in the 400–600 °C range). The char yield increases from 18.0 wt% for AB to 41.5 wt% for AB22.

**Figure 4:**
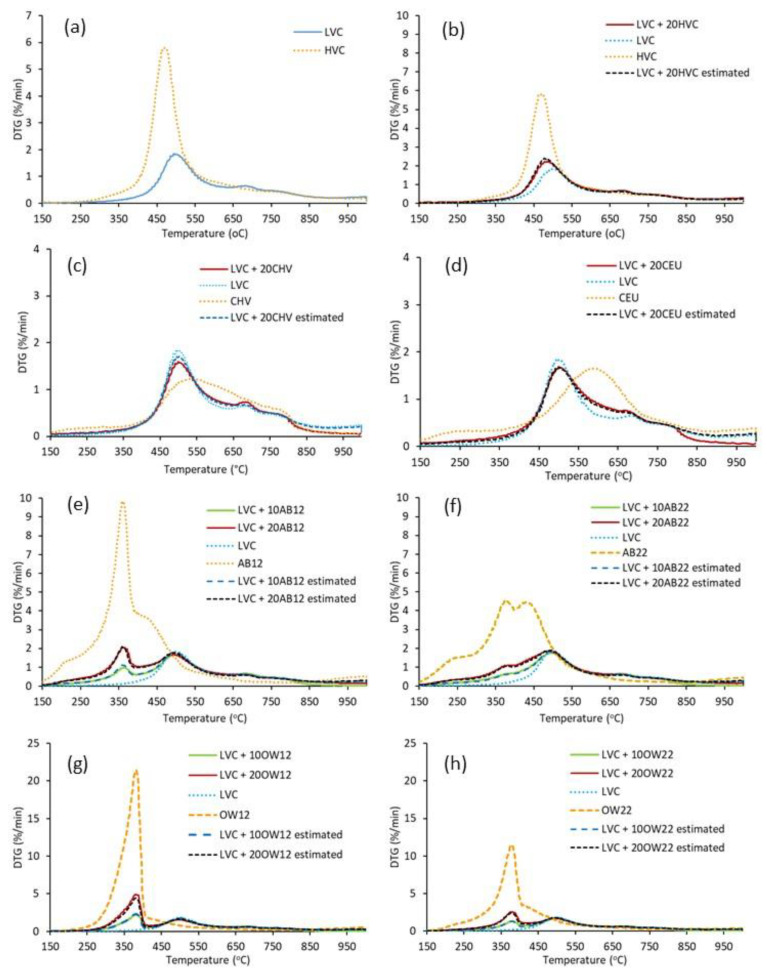
Experimental pyrolysis DTG profiles of LVC and HVC coal (**a**) and blends composed of the LVC coal with 20 wt% of HVC coal (**b**), HVC char (**c**) and eucalyptus char (**d**); and blends of the LVC coal with 10 and 20 wt% of AB12 (**e**), AB22 (**f**), OW12 (**g**) and OW22 (**h**). Profiles include the single components and those calculated by applying the additivity law.

Using biomass-derived products in PCI blends is a way to reduce CO_2_ emissions in iron and steel production, expand the range of low-grade materials that can be used as fuels and contribute significantly to reducing negative impacts associated with wastes at a local level. In this context, the interactions between coal and coal- and bio-based chars during co-pyrolysis have been studied to determine whether the components of binary blends interact in some way that causes a different effect than expected by applying the additivity law. Figure 4 shows the experimental DTG profiles of the single components, the binary blends, and the estimated DTG curves of the tested blends. Data derived from TG/DTG curves are summarized in Table S2. After blending the LVC coal with the HVC coal and the different chars at 10 and 20 wt%, the co-pyrolysis DTG curves depict different patterns according to the additive (Figure 4), where the devolatilization steps of the LVC coal and the hydrochars are distinguished. Regarding the influence of the waste amount added to LVC, it can be deduced that the higher the waste quantity, the higher the weight loss and the decomposition rate at the lowest temperature.

Independent thermal behavior is not the case for blends containing high-volatile coal, its char and the biochar from eucalyptus. The DTG patterns of these blends are associated with a great overlapping extent of the thermal breakdown of the organic coal matrix and the loss of the gaseous species of LVC coal. Thus, while adding 20 wt% of the high-volatile coal (HVC) to the high-rank coal increases the maximum degradation rate and lowers T_max_, both chars present the opposite effect (Table S2). However, any synergistic or antagonistic effects for char formation during co-pyrolysis could be discarded because the results clearly show a lack of interactions in the total weight loss during co-pyrolysis and, consequently, in the yield of the resultant char. Indeed, an excellent linear relationship between the experimental and calculated char yields at 600, 800 and 1000 °C indicates how well the additivity law predicts char yield, giving a coefficient of determination (R^2^) of 0.999 and a slope close to the unit (Figure S1). 

### 3.3. Combustion Behavior Using Thermogravimetry

**Figure 5:**
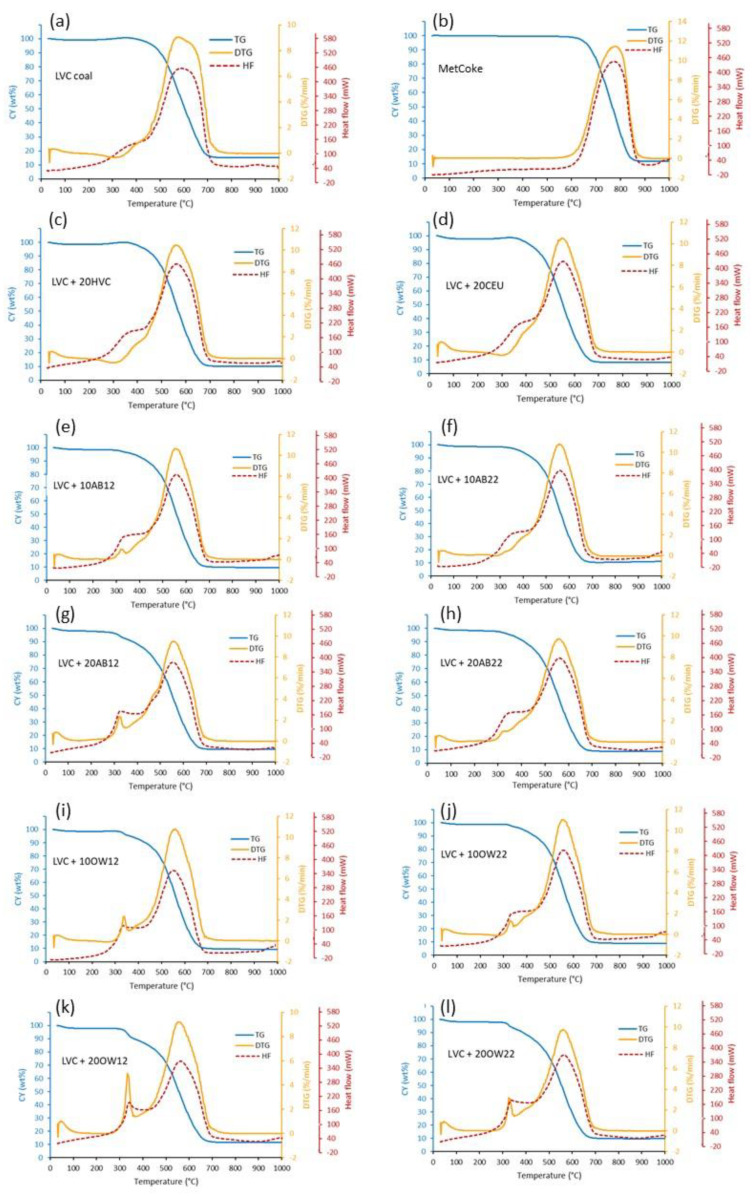
TG−DTG−HF co-combustion profiles of LVC (**a**), metallurgical coke (**b**) and blends with HVC at 20 wt% (**c**), CEU at 20 wt% (**d**), AB12 at 10 wt% (**e**), AB22 at 10 wt% (**f**), AB12 at 20 wt% (**g**), AB22 at 20 wt% (**h**), OW12 at 10 wt% (**i**), OW22 at 10 wt% (**j**), OW12 at 20 wt% (**k**) and OW22 at 20 wt% (**l**).

Non-isothermal combustion was performed to compare raw materials burning behavior and establish interactions, if any, between blend components. The TG-DTG-HF curves of single components and blends with LVC coal under the heating rate of 20 °C/min in an air atmosphere are shown in Figure S2 and Figure 5, respectively. One metallurgical coke with a moderate reactivity index (CRI) of 25.4% and high post-reaction strength (CSR) of 64.6% was also included for comparison purposes. Tables S3 and S4 present the burning characteristics of the candidate fuels, their blends with LVC and the metallurgical coke. After water removal, LVC coal and non-biomass additives increase in weight (about 1% of the initial sample weight) as the oxygen is adsorbed on some active sites of the carbon surface and heat is generated. The process starts at about 160 °C and continues to 360 °C, corresponding to O_2_ chemisorption [41,42,43], thus resulting in negative DTG values and exothermic heat. After forming unstable oxy-coal complexes, the combustion begins with a rapid weight decrease and increased heat generation (exothermic HF peak), ending when the organic matter is completely consumed.

The O_2_-chemisorption is not observed for biomass additives because devolatilization starts at this temperature, as seen by pyrolysis DTG curves (Figure 3). Next, the loss of inherent moisture and chemisorption, the combustion process proceeds via overlapping chemical reactions where the first combustion stage belongs to devolatilization with ignition and combustion of the volatile release up to 400 °C. Above 400 °C, the process is dominated by the ignition and combustion of the carbon contained in the char, which ends at a temperature above 700 °C for coals and coal-char and at a lower temperature for biomasic material. The DTG shape of the LVC coal combustion is rather broad, starting near 450 °C due to a small amount of volatiles and a residual inorganic material of about 15 wt% remaining after combustion. According to coal rank, the HVC coal used in PCI blends has more volatile matter than LVC, and earlier devolatilization can be observed with the subsequent heat generation (Table S3). 

Meanwhile, the DTG patterns of the derived hydrochars split into two distinct stages linked to the devolatilization of untransformed components during HTC with the combustion of volatile species and the rapid transition to ignition and combustion of the carbonaceous material. The total energy balance in both steps is exothermic, where energy is released as heat. The pattern of DTG-HF is quite similar, and the main differences lie in the first region corresponding to the devolatilization associated with pyrolysis, where volatiles evolve at a temperature below 400 °C, producing up to 50% of the total heat generated. Hydrochars produced at 230 °C have a higher thermal stability (T_50_) compared to hydrochars prepared at 180 °C, the highest combustibility range and the lowest reactivity (R_w_) due to a lower presence of the most reactive carbon and oxygen functionalities (Table S3). The combustion profiles are maintained when hydrochars are blended with the LVC coal, overlapping with the burning stage of the char from LVC coal at temperatures higher than 400 °C. The different additives sharpen the DTG profile and slightly decrease the T_max_ from 593 to around 575 °C (Figure 5, Table S4). 

**Figure 6:**
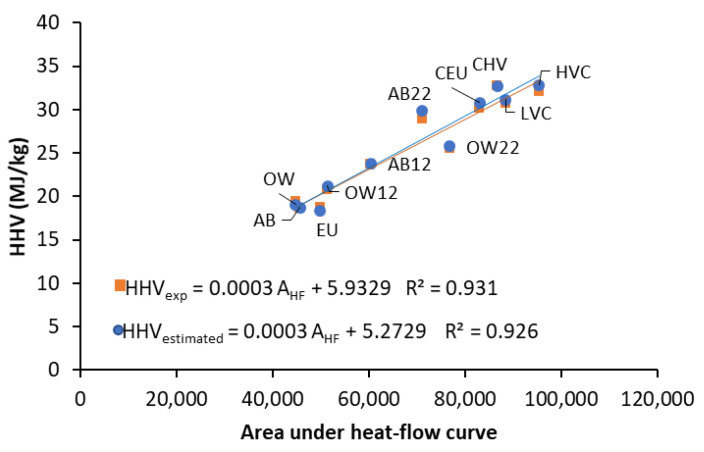
Relationships between the area under the heat flow curve (A_HF_) and the higher heating values experimentally obtained in a bomb calorimetric (HHV_exp_) and those estimated by the Channiwala and Parikh equation (HHV_estimated_) from elemental composition (C, H, N, S and O) and ash content (A) [27]. HHV_estimated_ = 0.3491C + 1.1783H + 0.1005S − 0.1034O − 0.0015N − 0.0211A.

As observed in the two-step combustion HF curves, when C and H combine with oxygen to give CO_2_ and H_2_O, more heat is released that is required for devolatilization. The area under the heat flow curve (A_HF_) represents the heat evolution during the oxidation reactions up to high temperature so that it could be related to the HHV of the sample, as determined with a bomb calorimeter. Figure 6 shows a positive linear relation of A_HF_ with HHVs of the coals, biomass and chars obtained using a bomb calorimeter. Some scatter may, however, be observed due to the different nature of the samples. Nevertheless, the experimental and predicted HHVs are relatively close, differing only in less than 4 MJ/kg. For confirmation purposes, Figure 6 also displays the HHV data calculated using the multiple regression model developed by Channiwala and Parikh [27], which is based on the elemental composition. Carbon and hydrogen are the primary elements of the organic matter of the fuels that contribute to the calorific value; other elements and ashes have no positive contribution or only contribute slightly.

Remarkably, the trend lines plotted in Figure 6 are practically the same, with less than 1 MJ/kg difference. Finally, the HHVs of the binary blends were estimated by applying the additivity law of the elemental composition and HHVs of their components and the values of the experimental heat flow during non-isothermal combustion (Table S5). To this end, the heat flow measured during combustion in a thermobalance can be considered an adequate alternative to the bomb calorimeter method and safely used to predict the calorific value. It can also be noticed that simultaneously to heat flow, several specific burning parameters in TGA can be obtained by examining the TG-DTG curves to estimate fuel combustion performance.

### 3.4. CO_2_-Gasification Behavior Using Thermogravimetry

**Figure 7:**
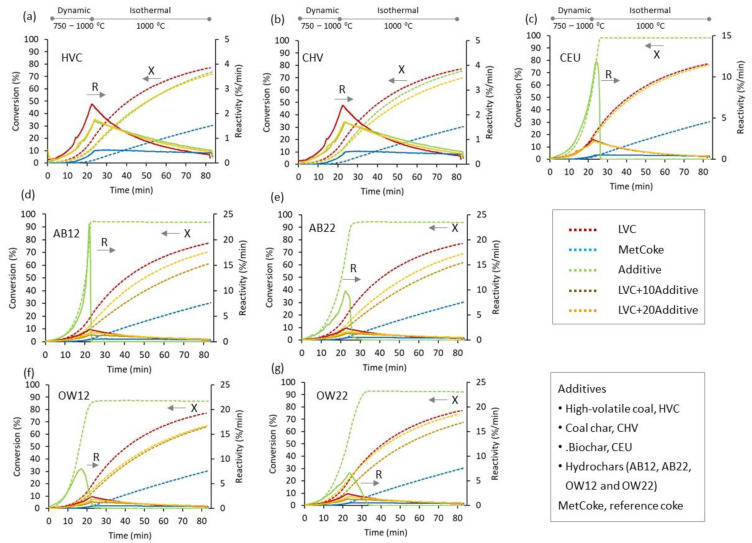
Evolution of the conversion degree (X, dashed lines) and reactivity to CO_2_ (R, solid lines) of the chars produced in situ in the thermobalance from LVC, single additives and the blends of LVC with: HVC (**a**), CHV (**b**), CEU (**c**), AB12 (**d**), AB22 (**e**), OW12 (**f**) and OW22 (**g**). Metallurgical coke added for reference.

From a qualitative point of view, the CO_2_-gasification profiles of the chars produced in situ at 800 °C from LVC and its blends with 10 and 20 wt% addition of coal and biomass during the previous pyrolysis step in the thermobalance seem to undergo a similar evolution. For comparison purposes, one metallurgical coke was also evaluated. The CO_2_-reactivity reaches a maximum value (R_max_) at 1000 °C at the end of the dynamic step (21–24 min), decreasing as the reaction progresses in the isothermal step at 1000 °C in the oven and tending towards stability with gasification time due to less available sites per residual mass and more resistant to the CO_2_ action. Such overall behavior obeys the well-known patterns of porous coal-based materials that depend on the access of CO_2_ to the internal surface area of the in situ chars and the presence of catalytic species [44,45]. Figure 7 compares the reactivity and conversion profiles of the chars, and Table S6 reflects the differences and similarities of the different blends and coke by the most relevant CO_2_-gasification parameters derived from the mass change during the dynamic and isothermal steps. Additionally, data corresponding to single additives are listed in Table S7. 

An examination of the reactivity and conversion degree of the chars produced in situ during pyrolysis at 800 °C reveals some remarkable features that reflect their response to the action of CO_2_. For instance, a remarkable similarity in the behavior of the coal chars produced in situ and by laboratory oven when blending with LVC (graphs marked in Figure 7 as HVC and CHV, respectively) can be observed. There is an overlap throughout the process with the char produced from the main component of the blend (LVC base-coal char) that dominates the gasification process. It also seems that the differences in the reactivity and conversion of the resultant chars are not very pronounced, since they have similar values at any degree (Table S5). On the contrary, metallurgical coke differs markedly from blends composed of coal- and biomass-derived chars. Because of its graphitizable carbon structure with fewer active sites participating in the C-CO_2_ reaction and less open structure, the high-temperature coke exhibits the lowest final conversion (X_f_ = 30.4% vs. 61.8–77.1%) and reactivity (R_max_ = 0.54 %/min *vs.* 1.30–2.38 %/min) under the conditions employed together to the highest gasification start temperature (T_initial_ = 929 °C *vs*. below 870 °C).

There are inherent differences when only biomass additives from the different parent materials are compared. The situation of the low-temperature eucalyptus char (CEU) is comparable to that of the out-of-use woods hydrochar prepared at 230 °C (OW22) blended at 20 wt%. The chars from these blends are the most reactive at any specific conversion degree; the onset temperature of the Boudouard reaction is nearly the same and close to the LVC coal, and they achieve a higher final conversion than the other chars. The OW22 hydrochar is greatly affected by its previous hydrothermal history at 230 °C where the degradation pathway of carbohydrates and reactive compounds during HTC induces a hydrochar with lignin, contributing to a greater extent to char formation at 800 °C. This finding is consistent with the pyrolysis behavior evidenced by TGA in Figure 4 (Section 3.2). With respect to this, the higher reactivity of LVC + OW22 due to a higher concentration of lignin than polysaccharides is in line with the variations found in the CO_2_-reactivity of high-temperature cokes obtained from blends of coking coals with a small addition of hemicellulose, cellulose, and lignin [16]. For high-temperature biocokes, the original biopolymers retain their structure but distortedly, and lignin has the most marked effect on coke reactivity, reaching the highest R_max_ in a shortest reaction time and the highest final conversion. When cellulose is added, biocoke exhibits an intermediate R_max,_ while xylan, a representative of hemicelluloses, has a moderate effect on coke reactivity [16,45]. However, other biomass wastes containing a high content of carbohydrates and a low content of lignin, like in blends with AB addition, give less-reactive chars. Regarding the increasing amount of the additive from 10 to 20% by weight, there is an increase in the degree of final conversion achieved (X_f_), a reduction in the threshold or starting temperature of gasification and those at low conversion degrees, resulting in more reactive chars. The significant reduction in reactivity can initially be explained by the relative amount of carbon formed from polysaccharides and lignin, since it must be an essential factor in controlling the formation of amorphous carbon and porous structure during the previous pyrolysis stage. In addition, it has been shown that hydrochars from apple bagasse at the two temperatures, 180 and 230 °C, lead to carbon forms less susceptible to being attacked by CO_2_.

## 4. Conclusions

This research highlights the importance of optimizing the chemical, compositional and thermal characteristics of raw biomass through thermochemical conversion processes to produce solid biofuels that can be effectively used in iron and steel production, contributing to reducing the use of fossil coal and CO_2_ emissions and promoting sustainable waste management practices. Additionally, the study provides valuable insights into the behavior of various PCI blends during the co-pyrolysis, co-combustion, and CO_2_-gasification of in situ chars, aiding in developing more efficient and environmentally friendly energy utilization strategies. No significant interactions affecting the char formation were found, while positive effects were mainly observed for higher conversion and a decrease in the ignition temperature of blends of coal and char under combustion conditions. Estimating the higher heating value of the blends using heat flow data was a fast and attractive strategy due to the strong exothermic reaction in combustion. Overall, the CO_2_-gasification of chars produced in situ from blends containing lignin-rich hydrochars exhibited higher reactivity and conversion than those with a higher carbohydrate content, making them more suitable for gasification applications. Thus, based on the observed effects caused by adding biomass products, secondary renewable fuels could be adjusted by an appropriate biomass selection and thermochemical conversion process to maintain blast furnace stability.

## Figures and Tables

**Table 1:** Main characteristics of the coals, biomass and coal- and bio-based chars.

Type of Material	Coals		Coal-Based Char	Woody Biomass	Biochar	Wastes		Hydrochars			
Code sample	LVC	HVC	CHV	EU	CEU	AB	OW	AB12	AB22	OW12	OW22
Moisture (wt%)	1.7	1.7	1.6	4.4	6.3	66.2	11.7	10.7	0.4	1.6	1.3
Ash (wt% db)	10.67	5.70	9.52	0.16	0.37	2.30	1.07	0.77	0.98	1.68	1.82
Volatile matter (wt% db)	18.4	37.8	20.5	83.7	30.4	79.0	81.6	70.1	56.1	76.7	61.8
Fixed carbon (wt% db)	70.9	56.5	70.0	16.1	69.2	18.7	17.3	29.1	42.9	21.6	36.4
C (wt% db)	78.57	78.60	80.83	46.73	81.64	46.02	47.76	59.39	71.61	52.06	62.59
H (wt% db)	3.75	5.37	4.25	5.88	3.24	6.25	5.80	5.47	5.90	6.04	5.75
N (wt% db)	2.19	1.46	1.92	0.36	0.21	0.49	2.88	1.61	1.69	1.34	2.76
S (wt% db)	0.23	0.65	0.60	0.04	0.10	0.10	0.04	0.10	0.05	0.03	0.06
Odif (wt% db)	4.59	8.22	2.88	46.83	14.44	44.84	42.45	32.66	19.77	38.85	27.02
HHV (MJ/kg)	30.7	32.1	32.7	18.7	30.2	18.6	19.4	23.7	28.9	20.8	25.5
LHV (MJ/kg)	29.9	30.1	31.8	17.5	29.5	17.3	18.2	22.6	27.7	19.5	24.3

**Table 2:** Ash composition of the coals and raw biomass.

Type of Material	Coals		Woody Biomass	Lignocellulosic Wastes	
Code Sample	LVC	HVC	EU	AB	OW
Na_2_O (%)	0.65	0.48	1.82	4.19	4.89
K_2_O (%)	1.90	2.02	1.97	38.30	3.15
CaO (%)	4.49	3.08	11.82	9.28	25.10
MgO (%)	2.06	1.57	3.99	8.01	3.09
Fe_2_O_3_ (%)	6.08	6.40	28.74	0.70	3.26
MnO (%)	0.10	0.06	nd	<0.001	0.29
P_2_O_5_ (%)	0.66	0.17	0.62	26.90	1.60
ZnO (%)	0.12	0.23	<0.10	0.27	0.27
Al_2_O_3_ (%)	23.02	22.25	18.96	1.15	6.44
SiO_2_ (%)	57.37	59.89	32.10	5.78	39.85
TiO_2_ (%)	0.24	1.06	0.26	<0.01	5.01
Basicity index -BI-	2.01	0.93	0.15	20.04	0.82

## Data Availability

Not applicable.

## Supplementary Materials

The following supporting information can be downloaded at: https://www.mdpi.com/article/10.3390/ma16196563/s1. Figure S1: Comparison between estimated yields with experimental data of the chars produced during co-pyrolysis of the binary blends at different temperatures, 600, 800 and 1000 °C. Char yields (CY) of binary blends obey the additivity law; Figure S2: TG-DTG-HF combustion profiles of single fuels; Table S1: Pyrolysis TG/DTG data of coals, biomass, and coal- and bio-based chars; Table S2: Pyrolysis TG/DTG data of the blends of the LVC coal with HVC, and coal- and bio-based chars; Table S3: Combustion characteristics of LVC and additives; Table S4: Combustion characteristics of LVC, its blends and a metallurgical coke; Table S5: Comparison of the estimated HHVs of the binary blends applying the equation of Channiwala and Parikh and the experimental heat flow during combustion at programmed temperature; Table S6: CO2 gasification characteristics of LVC, its blends and a metallurgical coke; Table S7: CO2 gasification characteristics of LVC and additives.
