# Supplementary material for: Exploring Hydrochars from Lignocellulosic Wastes as Secondary Carbon Fuels for Sustainable Steel Production
Source: Materials (Basel). 2023 Oct 5;16(19):6563. doi: 10.3390/ma16196563 (PMC10574071; doi:10.3390/ma16196563)
Supplement: Supplementary file 1 [file materials-16-06563-s001.zip › materials-2564037-supplementary.pdf]

Supplementary Materials

Exploring Hydrochars from Lignocellulosic Wastes as Secondary Carbon Fuels for Sustainable Steel Production

Álvaro Amado-Fierro, Teresa A. Centeno and María A. Diez*

Institute of Science and Technology of Carbon (INCAR), CSIC, Francisco Pintado Fe 26, 33011 Oviedo, Spain

*Correspondence: madiez@incar.csic.es

Supplementary information associated to the thermogravimetric evaluation of pyrolysis, combustion and CO_2-gasification of coals (HVC and LVC), lignocellulosic biomass (eucalyptus -EU-, apple bagasse -AB- and out-of-wood -OW-) and binary blends is as follows:

Table S1: Pyrolysis TG/DTG data of coals, biomass, and coal- and bio-based chars.

Table S2: Pyrolysis TG/DTG data of the blends of the LVC coal with HVC, and coal- and bio-based chars.

Table S3: Combustion characteristics of LVC and additives.

Table S4: Combustion characteristics of LVC, its blends and a metallurgical coke.

Table S5: Comparison of the estimated HHVs of the binary blends applying the equation of Channiwala and Parikh and the experimental heat flow during combustion at programmed temperature.

Table S6: CO_2 gasification characteristics of LVC, its blends and a metallurgical coke; Table S7. CO_2 gasification characteristics of LVC and additives.

Table S7. CO_2 gasification characteristics of LVC and additives.

Figure S1: Comparison between estimated yields with experimental data of the chars produced during co-pyrolysis of the binary blends at different temperatures, 600, 800 and 1000 °C. Char yields (CY) of binary blends obey the additivity law.

Figure S2: TG-DTG-HF combustion profiles of single fuels.

Identifier code of samples

- ***Coals:*** *high-volatile coal (HVC) and low-volatile coal (LVC).*
- ***Coal Char:*** *CHV, char produced from HVC at 450 °C.*
- ***Eucalyptus:*** *Raw woody biomass (EU) and the charcoal (CEU) produced at 450 °C.*
- ***Hydrochars:*** *The identification code of the resulting hydrochars consists of 4 alphanumeric characters, indicating the raw biowaste (AB, OW) followed by two numbers identifying the HTC temperature (1 and 2 for 180 and 230 °C, respectively) and the second digit (2) indicates the soaking time.*

Table S1. Pyrolysis TG/DTG data of coals, biomass, and coal- and bio-based chars.

Sample	**LVC**	**HVC**	**CHV**	**EU**	**CEU**	**AB**	**AB12**	**AB22**	**OW**	**OW12**	**OW22**
$VM_{200-300}$ (wt% db)	0.2	0.4	0.9	14.7	1.6	22.3	9.1	7.3	14.1	4.8	6.4
$VM_{300-400}$ (wt% db)	0.7	2.2	1.2	55.9	1.9	35.4	30.1	17.0	51.8	58.9	31.8
$VM_{400-600}$ (wt% db)	10.6	25.3	9.5	9.1	11.1	14.3	18.9	24.2	8.7	9.8	17.1
$VM_{600-800}$ (wt% db)	5.5	5.4	7.8	2.8	9.5	1.3	2.9	3.4	2.2	2.7	3.9
$VM_{800-1000}$ (wt% db)	2.4	2.2	**1.3**	**0.9**	3.7	3.0	3.5	3.0	2.2	3.7	2.9
CY_{600} (wt% db)	88.5	72.1	88.1	19.8	85.0	24.4	40.1	49.9	24.7	26.2	44.0
CY_{800} (wt% db)	83.0	66.7	80.3	17.0	75.5	23.0	37.2	46.5	22.5	23.5	40.1
CY_{1000} (wt% db)	80.5	64.4	79.0	16.1	71.8	20.1	33.6	43.6	20.3	19.8	37.2
T_i at X = 2% (°C)	342	324	211	241	210	182	192	190	218	262	220
T_f at X = 98% (°C)	1000	998	800	717	1000	1000	1000	1000	994	1000	1000
T_f - T_i (°C)	658	674	589	476	790	818	808	810	776	738	780
T_{max} (°C)	494	470	531	355	582	346	358	374/430[1]	372	382	374
DTG_{max} (%/min)	1.85	5.81	1.23	22.82	1.65	12.63	9.88	4.56/4.47[1]	14.92	21.43	11.46

[1]Partially overlapping double peak in the rate of volatile matter evolution.

Table S2. Pyrolysis TG/DTG data of the blends of the LVC coal with HVC, and coal- and bio-based chars.

Sample	**LVC**	**HVC**	**CHV**	**CEU**	**AB12**		**AB22**		**OW12**		**OW22**	
Addition rate (wt%)	-	20	20	20	10	20	10	20	10	20	10	20
$VM_{200-300}$ (wt% db)	0.2	0.4	0.4	0.6	1.0	2.2	0.9	1.4	0.7	0.8	0.8	1.5
$VM_{300-400}$ (wt% db)	0.7	1.1	0.8	1.1	3.3	7.2	2.4	3.6	6.4	7.9	3.9	7.5
$VM_{400-600}$ (wt% db)	10.6	13.3	10.0	11.2	11.1	12.3	11.9	13.6	10.4	11.0	11.3	12.3
$VM_{600-800}$ (wt% db)	5.5	5.9	6.2	6.3	5.5	5.2	5.5	10.2	5.5	5.4	5.5	5.3
$VM_{800-1000}$ (wt% db)	2.4	2.8	1.1	2.7	0.8	2.6	0.8	9.0	0.9	2.6	0.9	2.6
CY_{600} (wt% db)	88.5	85.1	88.6	86.9	84.4	77.9	84.6	81.1	82.4	80.1	83.9	78.4
CY_{800} (wt% db)	83.0	79.3	82.4	80.6	78.9	72.8	79.2	70.8	77.0	74.8	78.4	73.1
CY_{1000} (wt% db)	80.5	76.5	**81.3**	77.9	**78.1**	70.1	**78.3**	61.9	**76.0**	72.2	**77.5**	70.6
T_i at X = 2% (°C)	342	308	265	256	235	214	235	242	279	278	255	242
T_f at X = 98% (°C)	1000	1000	800	1000	800	1000	800	1000	800	1000	800	1000
T_f - T_i (°C)	658	692	535	744	565	786	565	758	521	722	545	758
T_{max} (°C)	494	478	499	502	363/491[1]	366/494[2]	379/491[2]	382[2]/478	379/499[1]	382/494[1]	379/499[1]	374/494[1]
DTG_{max} (%/min)	1.85	2.24	1.59	1.69	1.01/1.71[1]	2.19/1.67[2]	0.65/1.78[2]	0.95[2]/1.92	2.26/1.62[1]	2.71/1.70[1]	1.35/1.71[1]	2.56/1.70[1]

[1]Partially overlapping double peak in the rate of volatile matter evolution. [2]The low-temperature peak is a shoulder of the main DTG peak.

Table S3. Combustion characteristics of LVC and additives.

Sample	LVC	HVC	CHV	CEU	AB12	AB22	OW12	OW22
X_{n300} (%)[1]	-1.0	-1.1	0.1	1.0	19.1	11.0	7.3	10.1
X_{n350} (%)[1]	-1.8	-0.2	0.5	4.2	43.3	25.6	57.6	41.3
X_{n400} (%)[1]	-0.8	4.1	3.7	14.6	56.0	38.0	69.0	55.6
X_{n450} (%)	2.4	12.8	11.7	34.6	76.8	55.0	82.9	77.3
X_{n500} (%)	10.6	30.1	31.7	72.2	99.3	78.6	98.1	96.3
X_{n600} (%)	56.3	80.3	82.6	99.9	100.0	100.0	99.8	99.8
X_{n750} (%)	99.9	100.0	99.8	100.0	99.9	99.9	100.0	100.0
X_{n1000} (%)	100.0	100.0	100.0	100.0	100.0	100.0	100.0	100.0
CY_{1000} (wt% db)	15.37	7.17	7.72	0.55	3.59	3.32	1.64	1.68
$T_{ignition}$ at X_n = 2% db (°C)	444	380	380	326	202	210	260	232
T_{10} at X_n = 10% db (°C)	496	438	442	382	276	296	308	298
T_{20} at X_n = 20% db (°C)	528	470	474	416	300	328	322	310
T_{50} at X_n = 50% db (°C)	588	540	532	472	376	436	338	380
$T_{burnout}$ at X_n = 98% db (°C)	698	644	644	532	488	552	498	512
Combustibility range (°C)	230	252	250	200	276	330	222	260
T_{max} (°C)	574	458/550[2]	510	502	308/454[2]	310/486[2]	332/462[2]	314/452[2]
DTG_{max} (%/min)	9.05	6.33/9.70[2]	9.95	16.59	15.70/13.51[2]	6.83/9.41[2]	45.41/7.58[2]	23.60/9.92[2]
Rw x 10^7	1.0	1.8	1.9	5.5	14.2	7.0	24.8	15.7

[1]negative values in conversion (X) are linked to O_2-chemisorption; [2]two DTG peaks of different intensity and partially overlapped. X_n denotes normalized conversion at 100%.

Table S4. Combustion characteristics of LVC, its blends and a metallurgical coke.

Sample	**LVC**	**MetCoke**	**HVC**	**CHV**	**CEU**	**AB12**		**AB22**		**OW12**		**OW22**	
Addition rate (wt%)	-	-	20	20	20	10	20	10	20	10	20	10	20
X_{n300} (%)	-1.0	0.2	-1.1	-1.0	-0.8	0.5	1.9	0.3	1.1	-0.2	0.4	-0.2	0.5
X_{n350} (%)	-1.8	0.2	-1.7	-1.5	-0.9	2.7	6.6	1.4	3.6	3.1	7.8	2.0	5.6
X_{n400} (%)	-0.8	0.3	0.7	1.1	2.7	6.1	11.1	4.9	8.0	6.6	12.3	5.7	10.5
X_{n450} (%)	2.4	0.3	6.2	7.0	10.0	12.3	18.4	11.3	15.1	12.2	18.3	11.8	17.7
X_{n500} (%)	10.6	0.4	18.4	20.1	25.0	24.4	31.2	24.0	28.2	23.9	30.0	23.8	30.0
X_{n600} (%)	56.3	1.1	71.8	73.4	78.3	77.2	78.4	78.2	77.0	77.0	77.2	77.4	78.2
X_{n750} (%)	99.9	45.7	99.9	99.8	99.9	99.7	100.0	99.9	99.9	99.7	99.9	99.6	99.9
X_{n1000} (%)	100.0	100.0	100.0	100.0	100.0	100.0	100.0	100.0	100.0	100.0	100.0	100.0	100.0
CY_{1000} (wt% db)	15.37	11.75	10.15	9.19	8.35	9.80	9.70	11.35	8.93	9.52	11.75	8.98	9.99
$T_{ignition}$ at X_n = 2% db (°C)	444	624	414	414	392	332	300	362	322	338	322	348	322
T_{10} at X_n = 10% db (°C)	496	676	470	470	450	434	388	440	416	432	374	436	394
T_{20} at X_n = 20% db (°C)	528	702	504	504	486	484	456	488	472	486	460	488	462
T_{50} at X_n = 50% db (°C)	588	756	560	560	546	550	542	550	546	552	544	550	544
$T_{burnout}$ at X_n = 98% db (°C)	698	846	672	672	664	666	666	662	668	666	670	668	666
Combustibility range (°C)	238	210	242	242	256	316	348	282	328	310	328	300	326
T_{max} (°C)	574	774	558	558	550	558	558	558	558	558	558	558	558
DTG_{max} (%/min)	9.05	11.46	10.50	10.51	10.52	10.67	9.52	10.81	9.74	10.78	9.22	11.05	9.77
Rw x 10^7	1.0	0.6	1.5	1.6	1.8	2.5	2.7	2.1	2.4	2.4	2.2	2.3	2.4

X_n denotes normalized conversion at 100%.

Table S5. Comparison of the estimated HHVs of the binary blends applying the equation of Channiwala and Parikh and the experimental heat flow during combustion at programmed temperature.

Additive in the blend	**HVC**	**CHV**	**CEU**	**AB12**		**AB22**		**OW12**		**OW22**	
Addition rate (wt%)	20	20	20	10	20	10	20	10	20	10	20
HHV from elemental composition[1]	31.5	31.5	30.6	30.0	29.3	30.5	30.3	29.7	28.7	30.2	29.6
HHV from A_{HF}[2]	32.8	32.6	32.2	29.0	31.7	29.4	32.3	27.5	29.5	28.8	30.6

[1]The main elements (C, H, N, S and O) of the binary blends on dry basis were estimated from the data of the single components of the blends applying the law of additivity. Based on the elemental composition and ash content (A), the higher heating values (HHV) in (MJ/kg) were estimated using the equation of Channiwala and Parikh: $HHV_{estimated} = 0.3491C + 1.1783H + 0.1005S - 0.1034O - 0.0015N - 0.0211A$.

S.A. Channiwala, P.P. Parikh. A unified correlation for estimating HHV of solid, liquid and gaseous fuels. Fuel 81(8) 1051-1063 (**2002**). https://doi.org/10.1016/S0016-2361(01)00131-4.

[2]The good correlation between the HHV and the area under the heat flow (A_{HF}) in non-isothermal combustion of the single components $HHV_{estimated} = 0.0003\ A_{HF} + 5.2729$ ($R^2 = 0.926$) -see Figure 6- was using for estimating the HHV of the binary blends.

Table S6. CO_2 gasification characteristics of LVC, its blends and a metallurgical coke.

Sample	**LVC**	**MetCoke**	**HVC**	**CHV**	**CEU**	**AB12**		**AB22**		**OW12**		**OW22**	
Addition rate (wt%)	-	-	20	20	20	10	20	10	20	10	20	10	20
X_f (%)	77.1	30.4	72.2	69.8	73.1	60.9	70.5	61.8	68.9	66.1	66.8	67.4	74.2
X at R_{max} (%)	18.7	5.0	14.9	13.3	17.1	9.5	13.6	9.5	12.4	13.5	15.4	13.5	17.8
$X_{1000/0}$ (%)	18.1	2.4	14.1	12.9	16.7	9.3	13.2	9.3	12.1	13.2	15.1	13.3	17.0
$X_{1000/30}$ (%)	61.1	17.5	53.1	51.1	56.7	41.4	52.4	41.9	49.4	47.8	49.2	49.5	57.3
$X_{1000/60}$ (%)	77.1	30.4	72.2	69.8	73.1	60.9	70.5	61.8	68.9	66.1	66.8	67.4	74.2
$CY_{1000/0}$ (wt% db)	81.9	97.6	85.9	87.1	83.3	90.7	86.8	90.7	87.9	86.8	84.9	86.7	83.0
$CY_{1000/60}$ (wt% db)	22.9	69.6	27.8	30.2	26.9	39.1	29.5	38.2	31.1	33.9	33.2	32.6	25.8
T at R_{max} (°C)	986	988	987	987	987	986	987	986	987	986	986	986	986
$T_{initial}$ (°C)	852	929	829	856	852	869	859	871	871	851	844	856	844
T_{10} at X_n = 10% db (°C)	946	986	959	969	949	986	968	986	974	963	948	966	945
T_{20} at X_n = 20% db (°C)	1000	1000	1000	1000	1000	1000	1000	1000	1000	1000	1000	1000	989
t at R_{max} (min)	22.5	24.0	20.8	23.2	23.0	22.4	23.0	22.8	22.9	23.2	23.0	23.2	21.2
$t_{initial}$ (min)	7.4	17.6	7.4	9.4	8.9	10.1	9.7	10.7	9.9	9.1	8.0	9.5	6.2
t_{fin} (min)	77.8	81.9	80.5	79.7	78.9	79.6	79.3	80.0	79.6	79.9	79.5	79.7	77.0
Gasification time range (min)	70.4	64.3	73.1	70.3	70.0	69.5	69.6	69.3	69.7	70.8	71.5	70.2	70.8
t_{10} at X = 10% db (min)	17.9	31.1	20.7	21.0	18.9	22.7	20.8	23.1	21.3	20.5	18.7	20.8	16.6
t_{20} at X = 20% db (min)	23.0	51.9	27.0	27.2	24.4	30.9	26.6	31.1	27.7	27.6	26.1	27.2	22.3
t_{50} at X = 50% db (min)	41.0	-	50.4	51.7	45.3	63.4	50.1	62.8	53.3	55.7	53.8	53.6	43.1
R_{max} (%/min)	2.38	0.54	1.77	1.72	2.06	1.30	1.84	1.32	1.64	1.54	1.53	1.69	2.04
R_{10} at X = 10% db (%/min)	1.42	0.51	1.22	1.32	1.38	1.29	1.40	1.31	1.40	1.14	1.08	1.32	1.34
R_{20} at X = 20% db (%/min)	2.34	0.45	1.65	1.58	1.96	1.17	1.66	1.19	1.49	1.40	1.43	1.51	1.97
R_{50} at X = 50% db (%/min)	1.18	-	0.96	0.90	1.01	0.69	0.93	0.72	0.89	0.78	0.79	0.81	1.04

X denotes Conversion; X_n normalized conversion at 100 %; CY Char Yield; T Temperature; t Time; R Reactivity. 1000/0-30-60 denotes Conversion or Char Yield at 1000 °C and 0-30-60 min after reaching said temperature.

Table S7. CO_2 gasification characteristics of LVC and additives.

Sample	**LVC**	**HVC**	**CHV**	**CEU**	**AB12**	**AB22**	**OW12**	**OW22**
X_f (%)	77.1	73.8	75.5	98.2	94.0	94.3	87.3	92.9
X at R_{max} (%)	18.7	13.4	14.2	76.1	78.4	68.1	56.1	56.2
$X_{1000/0}$ (%)	18.0	13.0	13.5	73.5	94.0	77.2	86.1	57.1
$X_{1000/30}$ (%)	61.1	52.5	56.8	98.2	93.8	94.2	87.2	92.6
$X_{1000/60}$ (%)	77.1	73.8	75.5	98.2	93.6	93.9	86.8	92.3
$CY_{1000/0}$ (wt% db)	82.2	87.1	86.5	26.5	6.0	22.8	13.9	42.9
$CY_{1000/60}$ (wt% db)	22.9	26.2	24.5	1.8	6.4	5.1	13.2	7.7
T at R_{max} (°C)	986	987	987	985	976	978	924	986
$T_{initial}$ (°C)	852	850	842	813	801	810	790	811
T_{10} at X_n = 10% (°C)	946	968	965	879	885	862	842	875
T_{20} at X_n = 20% (°C)	988	988	987	913	917	897	870	912
T_{50} at X_n = 50% (°C)	1000	1000	1000	977	976	960	916	977
t at R_{max} (min)	22.5	21.5	23.2	24.0	22.1	22.5	16.9	23.3
$t_{initial}$ at X_n = 2% (min)	7.4	9.4	8.1	5.8	6.1	4.4	3.2	5.4
t_{fin} at X_n = 98% (min)	77.8	80.5	79.4	25.8	22.7	25.8	22.3	31.0
Gasification time range (min)	70.4	71.2	71.3	20.0	16.6	21.4	19.1	25.6
t_{10} at X = 10% db (min)	17.9	21.4	20.6	12.7	12.5	10.6	8.5	11.9
t_{20} at X = 20% db (min)	23.0	27.7	26.2	16.1	15.8	14.3	11.3	15.7
t_{50} at X = 50% db (min)	41.0	50.9	46.1	21.4	20.3	20.4	16.1	22.3
R_{max} (%/min)	2.38	1.72	1.95	11.83	23.11	9.83	8.02	6.63
R_{10} (%/min)	1.42	1.29	1.35	2.00	2.14	1.96	2.60	2.00
R_{20} (%/min)	2.34	1.57	1.89	3.77	3.98	3.20	4.46	3.32
R_{50} (%/min)	1.18	1.02	1.11	8.10	10.68	7.00	7.81	5.97

X denotes Conversion; X_n Normalized conversion at 100%; CY Char Yield; T Temperature; t Time; R Reactivity. 1000/0-30-60 denotes Conversion or Char Yield at 1000 °C and 0-30-60 min after reaching said temperature.

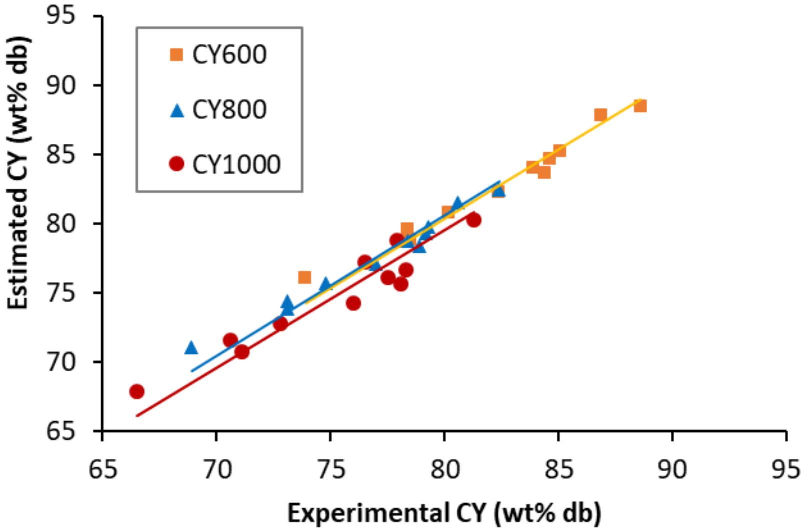

Equation	Coefficient of determination
CY_{600} estimated = 1.0045 CY_{600} experimental	R^2 = 0.9999
CY_{800} estimated = 1.0074 CY_{800} experimental	R^2 = 0.9999
CY_{1000} estimated = 0.9940 CY_{1000} experimental	R^2 = 0.9997

Figure S1. Comparison between estimated yields with experimental data of the chars produced during co-pyrolysis of the binary blends at different temperatures, 600, 800 and 1000 °C. Char yields (CY) of binary blends obey the additivity law.

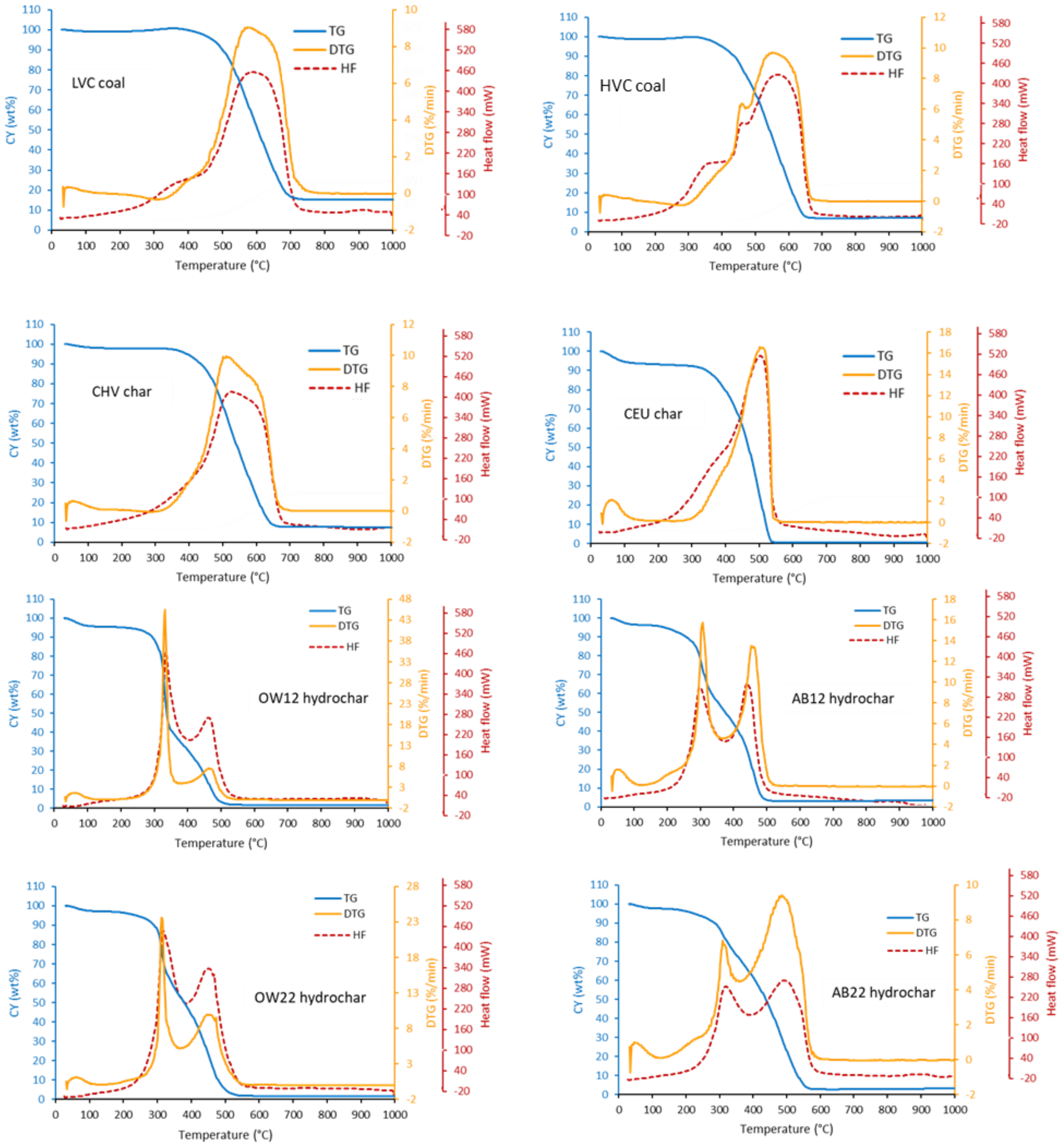

Figure S2. TG-DTG-HF combustion profiles of single fuels.
